# *Drosophila* nucleostemin 3 is required to maintain larval neuroblast proliferation

**DOI:** 10.1016/j.ydbio.2018.04.014

**Published:** 2018-04-19

**Authors:** Patrick W. Johnson, Chris Q. Doe, Sen-Lin Lai

**Affiliations:** Howard Hughes Medical Institute, Institute of Neuroscience, Institute of Molecular Biology, Department of Biology, University of Oregon, Eugene, OR 97403, USA

**Keywords:** Nucleostemin, Prospero, Neuroblast, Subcellular localization, Proliferation

## Abstract

Stem cells must maintain proliferation during tissue development, repair and homeostasis, yet avoid tumor formation. In *Drosophila*, neural stem cells (neuroblasts) maintain proliferation during embryonic and larval development and terminate cell cycle during metamorphosis. An important question for understanding how tissues are generated and maintained is: what regulates stem cell proliferation versus differentiation? We performed a genetic screen which identified *nucleostemin 3* (*ns3*) as a gene required to maintain neuroblast proliferation. *ns3* is evolutionarily conserved with yeast and human Lsg1, which encode putative GTPases and are essential for organism growth and viability. We found NS3 is cytoplasmic and it is required to retain the cell cycle repressor Prospero in neuroblast cytoplasm via a Ran-independent pathway. NS3 is also required for proper neuroblast cell polarity and asymmetric cell division. Structure-function analysis further shows that the GTP-binding domain and acidic domain are required for NS3 function in neuroblast proliferation. We conclude NS3 has novel roles in regulating neuroblast cell polarity and proliferation.

## 1. Introduction

Stem cells have the unique capacity to maintain their undifferentiated state while rapidly producing lineage-specific, differentiating daughter cells. Towards the end of development, stem cells may enter a reversible, non-dividing G_0_ state, known as quiescence, or permanently exit cell cycle to undergo differentiation, senescence, or apoptosis. Determining the mechanisms that maintain stem cell proliferation may help illuminate mechanisms that impair development or cause tumor-igenesis.

*Drosophila* neuroblasts (NBs) have emerged as a widely used model system for studying stem cell proliferation, self-renewal, and cell cycle exit (Doe, 2008; Egger et al., 2008; Homem and Knoblich, 2012; Homem et al., 2015; Maurange and Gould, 2005). NBs delaminate from the neuroectoderm and immediately proceed to undergo asymmetric cell division to self-renew and generate neurons or glia. Most NBs in the thorax and central brain enter quiescence at the end of embryogenesis, and subsequently re-enter the cell cycle and proliferate after larval hatching. Based on their cell lineage, two types of NBs are distinguishable within the nervous system: type I and type II. The majority of NBs are categorized as type I NBs, which asymmetrically divide to self-renew and produce a smaller ganglion mother cell (GMC) that further divides to produce two neurons or glia. Type I NBs can also switch to type 0 during late embryonic development to produce a single neuronal daughter cell, instead of a GMC (Baumgardt et al., 2014, 2009; Bertet et al., 2014; Karcavich and Doe, 2004). In addition, there are eight type II NBs in each bilateral brain lobe that asymmetrically divide to self-renew and generate a smaller intermediate neural progenitors (INPs) that each undergo multiple rounds of asymmetric cell division to self-renew and produce GMCs. The latter type of division allows NBs to rapidly expand their lineage within a limited proliferation window (Bayraktar et al., 2010; Bello et al., 2008; Boone and Doe, 2008; Bowman et al., 2008; Walsh and Doe, 2017).

Proliferative embryonic NBs contain a set of transcription factors that promote stem cell attributes (asymmetric cell division, self-renewal, survival, and proliferation); this set of factors include the basic helix-loop-helix transcription factors Deadpan (Dpn) and Asense (Ase), the Snail family gene Worniu (Wor), the cell cycle regulator Cyclin E (CycE), the Sox family members SoxN and Dichaete (D), and the early temporal genes Hunchback (Hb), Krupple (Kr), and POU domain protein family genes (Nub/Pdm2) (Bahrampour et al., 2017; Lai and Doe, 2014; Lai et al., 2012). Proliferative embryonic and larval type I NBs also express the differentiation-promoting Prospero (Pros) transcription factor, but Pros protein is not active due to its segregation into the cytoplasm. Pros is an atypical homeodomain transcription factor with similar functions to its mammalian ortholog, Prox1, to repress progenitor-specific genes, repress cell cycle genes, and promote cell cycle exit and differentiation (Doe et al., 1991; Dyer et al., 2003; Elsir et al., 2012; Vaessin et al., 1991). Although high levels of nuclear Pros triggers cell cycle exit and differentiation (Bayraktar et al., 2010), low levels of Pros induce NB quiescence via repression of *ase*, *wor*, and *cycE*, but not *dpn* (Lai and Doe, 2014). NB cortical polarity is also essential for maintaining proliferation. The atypical protein kinase C (aPKC) is localized to the NB apical cortex and is required to restrict the Miranda/Pros complex to the basal cortex, where it is partitioned into the newborn GMC at mitosis (Atwood and Prehoda, 2009). Loss of aPKC results in delocalization of Miranda/Pros from the cortex, nuclear import of Pros, and NB cell cycle arrest (Rolls et al., 2003).

Here we use a forward genetic screen to identify essential genes that are expressed in NBs and required to maintain NB proliferation. We identify the highly conserved Lsg1 family gene *nucleostemin 3* (*ns3*) as being required for maintaining larval NB proliferation. Moreover, we find that cytoplasmic retention of Pros is regulated by NS3, independent of Ran-mediated nucleo-cytoplasmic trafficking, and knocking down *ns3* leads to the disruption of NB asymmetric cell division. We further show that NS3 requires the GTP-binding domain to be excluded from the nucleus, and the GTP-binding domain and acidic domain act together to retain Pros in the cytoplasm.

## 2. Materials and methods

### 2.1. Fly stocks

The following flies were used: (1) *wor-gal4* (Lai et al., 2012); (2) *wor-gal4, ase-gal80, UAS‐mCD8:GFP* (Carney et al., 2013; Joy et al., 2015); (3) *UAS-Dcr-2; wor-gal4; UAS‐mCD8:GFP* (Carney et al., 2013; Joy et al., 2015); (4) *UAS-nls-nes*^*+*^*-gfp* (FBst0007032); (5) *UAS-ns3-RNAi* (FBst0036626); (6) *UAS-pros-RNAi* (FBst0042538); (7) *tub-Gal80*^*ts*^ (FBst0007019). RNAi lines used in the screening experiments are listed in Table S1.

### 2.2. NS3 rescue constructs

*UAS‐HA:ns3*^*WT*^, *UAS‐HA:ns3*^*ΔA*^, *UAS‐HA:ns3*^*ΔG*^ and *UAS‐HA:ns3*^*ΔB*^ were generated by using PCR to amplify each individual fragment which was later assembled directly in *Eco*RI/*Not*I digested *pUAST-attB* with NEBuilder (New England BioLabs, Ipswich, MA). Primers used in PCR and fragments used for each construct are listed in Table S2. All constructs were verified by sequencing (Sequetech, Mountain View, CA) and inserted into *attP40* (FBst0036304) via PhiC31 integrase-mediated transgenesis (BestGene Inc, Chino Hills, CA). To prevent RNAi knockdown of the rescue constructs, we mutated the DNA sequence in the acidic domain targeted by *ns3* RNAi from GCCAGGTATGTGCTTAAAGACTAC to GCACGATACGTATTAAAGGATTAC.

### 2.3. Dissections, antibody staining, fixation and confocal microscopy

The following primary antibodies were used: rabbit anti-atypical PKC (1:1000) (Santa Cruz Biotechnology), rabbit anti-Asense (Cheng-Yu Lee, Univ. of Michigan, Ann Arbor, MI), rat anti-Dpn (1:100) (Abcam), guinea pig anti-Dpn (1:2000) (Jim Skeath, Washington Univ., St Louis, MO), mouse anti-Elav (1:50) (9F8A9, DSHB), chicken anti-GFP (1:500) (Aves Labs), mouse anti-Lamin (ADL67.10, DSHB), guinea pig anti-Miranda (1:500) (Doe lab), rabbit anti-PH3 (EMD Millipore), mouse anti-Pros purified antibody (1:500) (MR1A, Abcam), and rat anti-Worniu (1:100) (Abcam). Fluorophore-conjugated secondary antibodies were from Jackson ImmunoResearch Laboratories (West Grove, PA). Texas Red^™^-X Phalloidin (ThermoFisher Scientific) and DAPI (Sigma-Aldrich) were applied after secondary antibody staining by following manufacturer’s protocol. Larvae were raised at 29°C until third instar, and the brains were dissected, fixed and stained by following published procedures (Lai and Doe, 2014; Lai et al., 2012). Images were taken with Zeiss confocal microscope LSM710, processed with open source software Fiji (Schindelin et al., 2012) and assembled in the software Adobe Photoshop or Illustrator (Adobe Systems Inc., San Jose, CA). All statistical analyses in the figures used one-tail *t*-test included in Microsoft Excel (Microsoft Corp., Redmond, WA).

### 2.4. EdU incorporation

Immediately following dissections, brains were incubated in PBS containing 100 μg/mL EdU (ThermoFisher Scientific) at room temperature for 2hr with rocking. After standard fixation and antibody staining, EdU was detected by following the manufacturer’s protocol.

### 2.5. Cellular localization of NLS-NES analysis

We used the “Plot Profile” functionality of Fiji to measure the intensity of GFP-NLS-NES and Deadpan signal across the diameter of 5 NBs, whose cell cortex was outlined by Phalloidin staining. We then used R software to normalize the intensity and NB length to the scale 0–1, and smoothing splines were fit to the 5 data sets for each genotype. The R code is available upon request.

## 3. Results

### 3.1. An RNAi screen identifies genes required to maintain larval neuroblast proliferation

We used an RNAi-based approach to screen for genes that, when knocked down in larval brains, led to a loss of larval NB proliferation without decreasing NB numbers (i.e. specifically leading to quiescence or permanent cell cycle arrest). We screened 28 essential genes that we previously found to have enriched expression in NBs (Carney et al., 2012). For each gene, we used the NB-specific *worniu-gal4* transgene to drive expressions of a single copy of each *UAS-RNAi* transgene in NBs and membrane-tethered GFP to mark the affected NB lineage. NBs were identified by staining for Deadpan (Dpn), which marks both proliferating and quiescent NBs (Lai and Doe, 2014), and we assessed NB cell cycle progression via a 2-hr pulse of EdU.

**Fig. 1.**
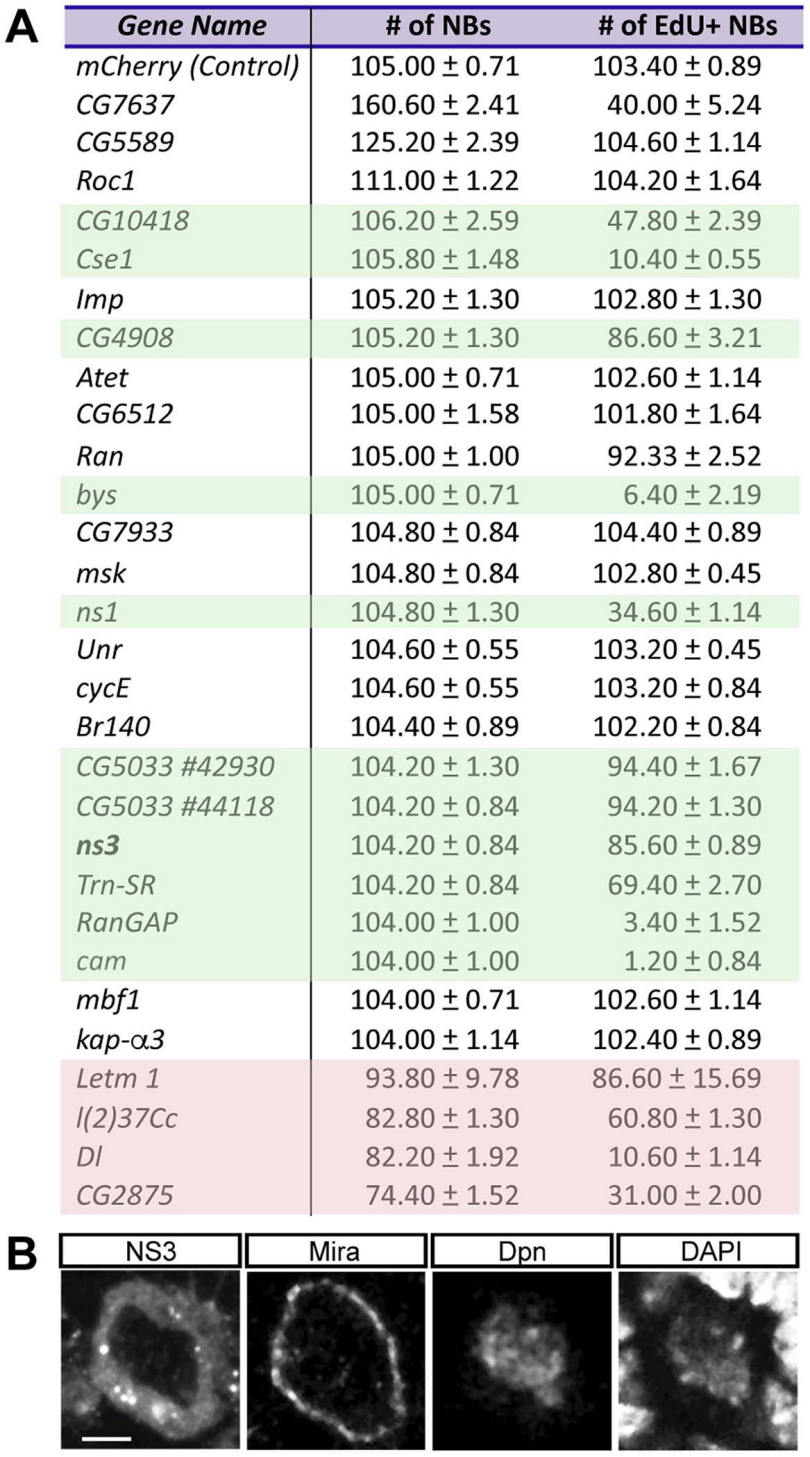
RNAi screen identifies candidate genes to promote NB proliferation. (A) The 28 NB-enriched genes assayed for RNAi phenotypes. The number of total NBs and EdU+ NBs in the L3 brain were quantified for each RNAi gene knockdown. Green shading indicates RNAi lines where no significant change in NB number (p > 0.05) but a decrease in EdU+ NBs (p < 0.05) indicating premature NB cell cycle exit. Red shading indicates RNAi lines that lead to a reduction in total NB number, due to NB death or differentiation. n = 5 brain lobes from 5 larvae for each gene. Genotype: *UAS-Dcr-2; wor-gal4; UAS‐mCD8:GFP/UAS‐gene RNAi*. (B) A representative neuroblast in the L3 brain. *wor-gal4* was used to drive expression of *UAS‐ns3:YFP* (FBst0050769). The neuroblast was stained with membrane marker Mira, NB marker Dpn, and DNA marker DAPI. Scale bar: 5 μm. Genotype: *wor‐gal4/UAS‐ns3:YFP*.

In wild type larval brains there are ~105 NBs per lobe, and all are labeled by a 2hr pulse of EdU (Fig. 1A, top row). In contrast, knocking down expression of 11 genes led to a significant reduction in EdU + NBs without decreasing NB number (Fig. 1A, green highlighted); this represents induction of NB cell cycle exit. Knockdown of an additional 4 genes showed reduced NB number (Fig. 1A, red shading); these are likely to be genes required to prevent NB death or differentiation, and while interesting, we do not focus on this class of genes here. We conclude that loss of function of 11 genes results in NB cell cycle exit without inducing differentiation or death.

Among the 11 candidate genes, we were particularly interested in the conserved Lsg1 family member, called *nucleostemin 3* (*ns3*) in *Drosophila*. The yeast and human ortholog, *Lsg1*, is cytoplasmic and promotes release of the nuclear export adapter from the large ribosomal subunit for ribosome maturation (Hedges et al., 2005; Kallstrom et al., 2003; Reynaud et al., 2005; Weis et al., 2014). In *Drosophila*, NS3 is also cytoplasmic in all cell types examined including larval NBs (Fig. 1B), and required in serotonergic neurons to regulate organismal growth (Hartl et al., 2013; Kaplan et al., 2008), yet little is known its roles in *Drosophila* nervous system development. Here, we investigate the role of NS3 in regulating NB proliferation.

### 3.2. Reduced NS3 leads to proliferation arrest in type I NBs

**Fig. 2.**
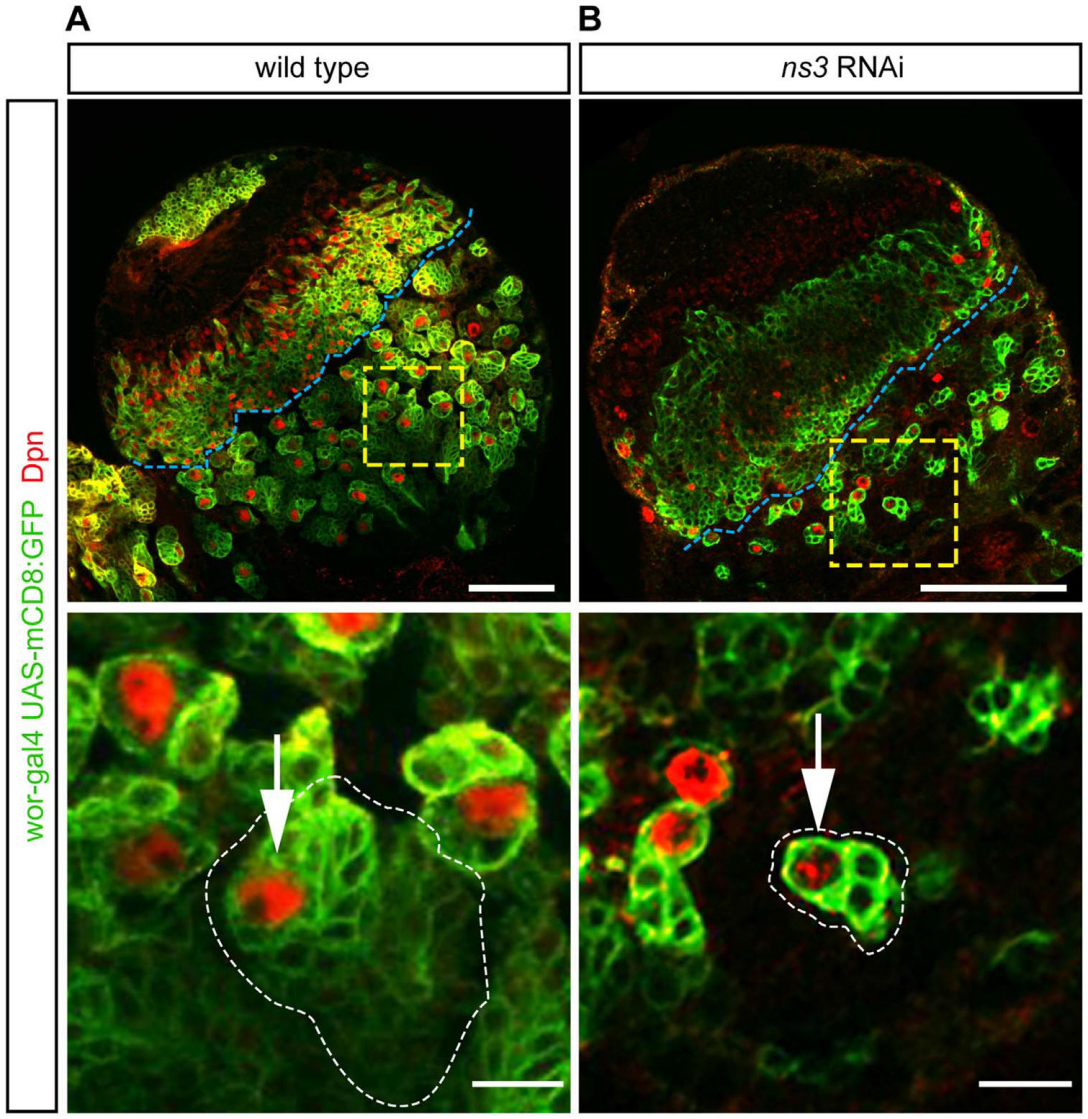
*ns3* RNAi mutant NBs in the central brain have reduced lineage sizes. Single slice confocal images of L3 wild type and RNAi brain lobes; NBs are identified by Dpn and NBs and their lineages are marked with GFP (*wor-gal4, UAS-mCD8:GFP*). (A) Wild type NBs produce large clones of neuronal progeny. Genotype: *UAS-Dcr-2; wor-gal4; UAS-mCD8:GFP*/*UAS-mCherry-RNAi*. (B) *ns3* RNAi NBs show reduced numbers of neuronal progeny. Genotype: *UAS-Dcr-2; wor-gal4; UAS-mCD8:GFP/UAS-ns3-RNAi*. The central brain / optic lobe boundary is indicated by a blue line. Representative NBs (white arrows) are boxed in yellow and enlarged in the bottom row. Scale bars: 50 μm top row, 10 μm bottom row.

Wild type larval NBs produce a clone of neuronal progeny that can be visualized by expression of GFP in the NB (*worniu-gal4 UAS‐ mCD8:GFP*) followed by perdurance of the GFP in the most recently born progeny (Fig. 2A, green). We found that *ns3* RNAi NBs had fewer neuronal progeny (Fig. 2B), despite normal NB numbers. Loss of NS3 could lead to NB cell cycle arrest or quiescence; the persistence of Dpn^+^ NBs rules out apoptosis or differentiation.

**Fig. 3.**
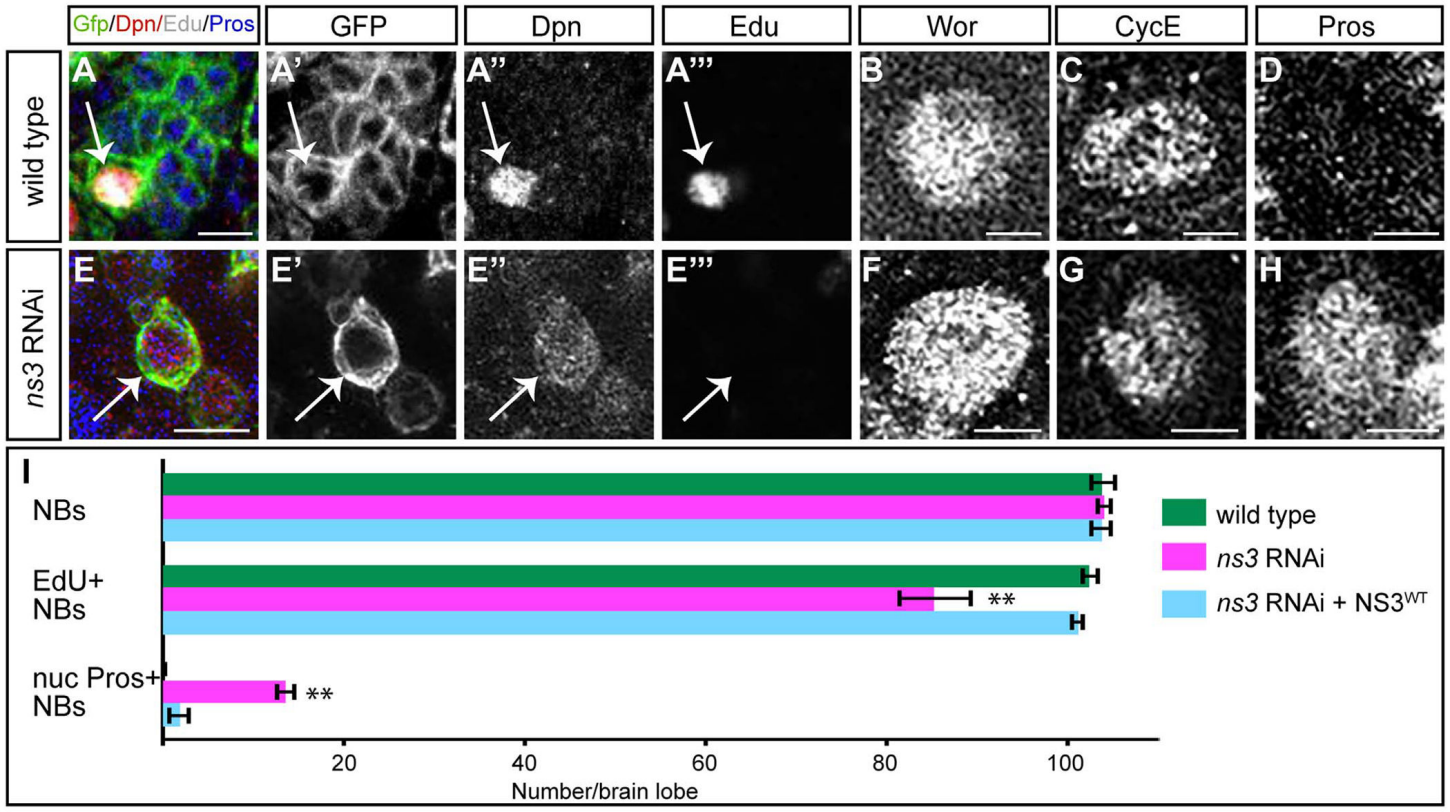
Reduced NS3 leads to proliferation arrest in type I NBs. (A-D) Wildtype type I NBs (white arrows) in the L3 brain, identified by *wor-gal4 UAS- mCD8:GFP* (A′) and Dpn (A”). They can be labeled by a pulse of EdU (A′”), are Wor^+^ (B) and CycE^+^ (C), and have no detectable nuclear Pros (D). The Wor, CycE and Pros images were obtained from different confocal stacks. Genotype: *UAS-Dcr-2; wor-gal4; UAS-mCD8:GFP/UAS-mCherry-RNAi*. (E-H) *ns3* RNAi type I NBs (white arrows) in the L3 brain, identified by *wor-gal4 UAS- mCD8:GFP* (E′) and Dpn (E”). They are not labeled by a pulse of EdU (E′”), but are Wor^+^ (F) and CycE^+^(G), and have nuclear Pros (H). The Wor, CycE and Pros images were obtained from different confocal stacks. Genotype: *UAS-Dcr-2; wor-gal4; UAS-mCD8:GFP/UAS-ns3-RNAi*. (I) Quantifications. n = 5 brain lobes. Error bars indicate s.d. * *: p < 0.001. Scale bars: 10 μm (A, E) and 5 μm (B-D, F-H).

We used cell cycle progression, molecular markers, and morphological criteria to determine whether reduced NS3 resulted in NB cell cycle arrest or quiescence. Wild type NBs can be labeled by a pulse of EdU, are Dpn^+^ Wor^+^ CycE^+^, and lack nuclear Pros (Fig. 3A–D). In contrast, quiescent NBs are Dpn^+^ Wor^−^ CycE^−^ nuclear Pros^+^, and extend a neurite-like process (Chell and Brand, 2010; Lai and Doe, 2014; Tsuji et al., 2008). We found that *ns3* RNAi NBs did not match either profile. They did not incorporate EdU like quiescent NBs but were Dpn^+^ Wor^+^ CycE^+^ nuclear Pros^+^ (Fig. 3E–H), but they failed to extend a neurite-like process (Fig. 2B, 3E′). These results suggest that reduced NS3 leads to NB cell cycle arrest, but not entry into quiescence. Importantly, overexpression of full length NS3 can fully rescue the *ns3* RNAi phenotype (Fig. 3I), showing NS3 is responsible for the cell cycle arrest phenotype.

To further test whether *ns3* RNAi leads to larval NB proliferation arrest or quiescence, we transiently knocked down NS3 and assayed for NB arrest followed by NB re-entry into the cell cycle, a hallmark of quiescent NBs. We used temperature-sensitive Gal80 (Gal80^ts^) to temporally control the expression of Gal4-induced *ns3* RNAi. We raised the *ns3* RNAi larvae for two days at 29°C (where Gal80^ts^ is inactive) to allow expression of *ns3* RNAi to induce NB proliferation arrest, followed by 2 days at 23°C (where Gal80^ts^ is active) to restore NS3 levels. We found that NBs remain cell cycle arrested (26.0 ± 2.9 NBs, *n* = 6 brain lobes), despite recovery of NS3 expression. We conclude that permanent or transient reduction of NS3 in type I NBs results in NB cell cycle arrest, but not entry into quiescence.

### 3.3. NS3 promotes Pros nuclear export in type I NBs through a Ran-independent mechanism

We noted that *ns3* RNAi NBs had nuclear Pros, which has been shown to arrest the NB cell cycle (Lai and Doe, 2014). This led us to investigate how NS3 promotes Pros nuclear export. Pros contains a canonical nuclear localization signal (NLS) to drive nuclear import (Hirata et al., 1995). Additionally, Pros harbors a nuclear export signal (NES) near its conserved homeodomain (Demidenko et al., 2001). The competition between NLS-driven import and NES-driven export determines the subcellular localization of Pros, however, the underlying mechanisms remain unclear (Bi et al., 2005, 2003; Demidenko et al., 2001; Ryter et al., 2002).

Most NLS/NES containing proteins require Ran-dependent transport across the nuclear membrane (Terry et al., 2007; Yudin and Fainzilber, 2009). Ran cycling is expedited by a steep concentration gradient between cytoplasmic Ran GTPase activating protein (RanGAP) and nuclear RanGTP exchange factor (RanGEF) (Steggerda and Paschal, 2002). In *Drosophila*, Ran, RanGAP, and RanGEF (Bj1) are all enriched in NBs (Carney et al., 2013; Joy et al., 2015), and the loss of any of these genes induces Pros nuclear localization (Joy et al., 2015, data not shown). Moreover, knocking down Ran or RanGAP also halted cell cycle progression without altering the quantity of NBs (Joy et al., 2015; Fig. 1B). These results led us to investigate whether NS3 has a novel role in Ran-mediated nuclear transport and functions together with Ran to control the nuclear-cytoplasmic localization of proteins with both an NLS and NES, including Pros.

**Fig. 4.**
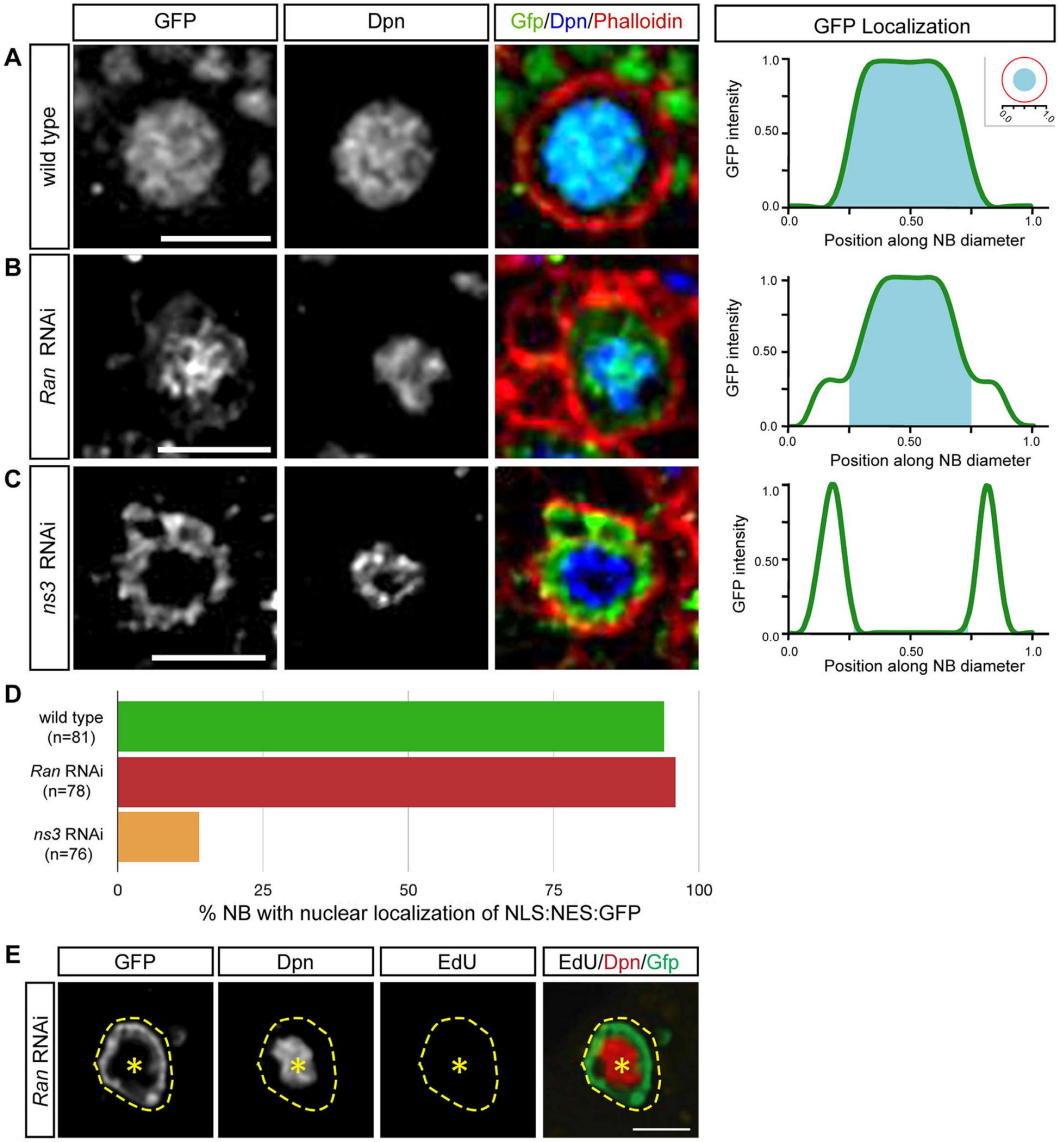
NS3 regulates Pros localization via a Ran-independent pathway. (A-D) Localization of NLS:NES:GFP (green line) in wild type (A), *Ran* RNAi (B), and *ns3* RNAi (C) L3 brain NBs. The NB nucleus marked by Dpn (cyan area); pixel intensity trace in right panels. Quantification (D) from 5 brains, n = number of NBs. Genotype: (A) *wor-gal4 UAS-NLS:NES:GFP; UAS-mCherry-RNAi*; (B) *wor-gal4 UAS-NLS:NES:GFP; UAS-Ran-RNAi*; (C) *wor-gal4 UAS-NLS:NES:GFP; UAS-ns3-RNAi*. Scale bars: 10 μm (E) Confocal images of a *Ran* RNAi L2 central brain NB lineage; NB and its lineage are marked with GFP (*wor-gal4, UAS-mCD8:GFP*), and NB is identified by Dpn. NB is EdU-negative. Genotype: (E) *UAS-Dcr-2; wor-gal4; UAS-mCD8:GFP/UAS-Ran-RNAi*. Scale bars: 10 μm.

To test this hypothesis, we determined the role of NS3 and Ran in the nucleo-cytoplasmic localization of a dual NLS/NES GFP reporter, NLS: NES:GFP (Kusano et al., 2001). In wild type NBs, NLS: NES:GFP was enriched in the nucleus, showing that the NLS was dominant over the NES (Fig. 4A, quantified in D). Similarly, *Ran* RNAi NBs showed nuclear localization of NLS: NES:GFP, showing that Ran was not required for nuclear localization of NLS: NES:GFP (Fig. 4B, quantified in D). In contrast, *ns3* RNAi larval NBs localized NLS: NES:GFP to the cytoplasm, showing that NS3 was required for nuclear import of the NLS: NES:GFP protein (Fig. 4C, quantified in D). We can be sure the *Ran* RNAi was effective, because it arrested the NB cell cycle (Fig. 4E). Interestingly, this is the opposite of the role of NS3 in preventing nuclear localization of Pros (see Discussion). Nevertheless, the finding that Ran RNAi and the NS3 RNAi had opposite phenotypes in this assay strongly suggests that NS3 is acting in in a Ran-independent pathway.

### 3.4. Reduced NS3 leads to proliferation arrest in Prospero-negative type II NBs

**Fig. 5.**
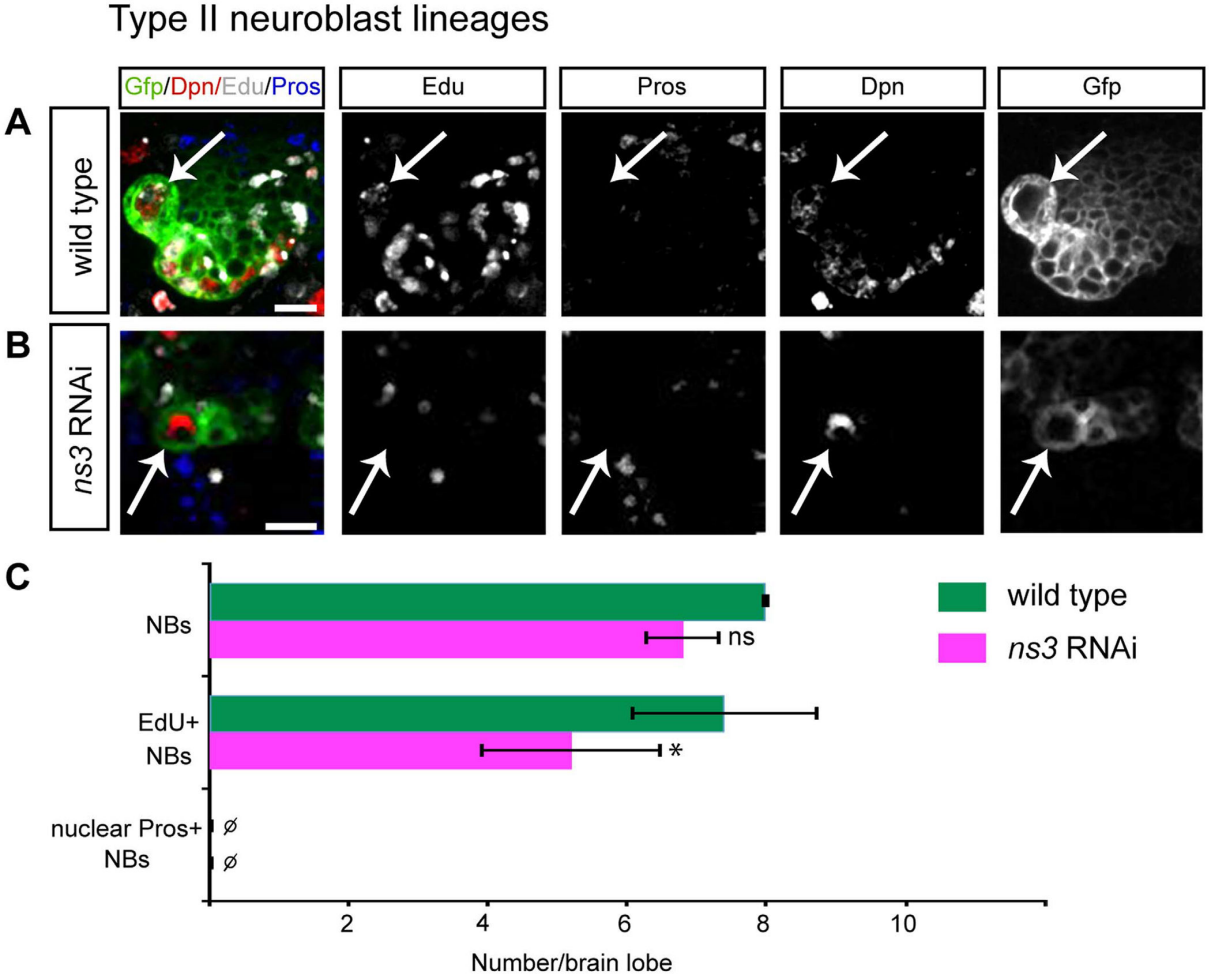
Reduced NS3 leads to proliferation arrest in type II NBs. Type II NBs (white arrows) in the L3 brain, identified by Dpn and *wor-gal4 ase-gal80 UAS-GFP*. (A) Wild type NBs are labeled by a pulse of EdU. Genotype: *UAS-Dcr-2; wor-gal4 ase-gal80; UAS-mCD8:GFP/UAS-mCherry-RNAi*. (B) *ns3* RNAi NBs are not labeled by a pulse of EdU. Genotype: *UAS-Dcr-2; wor-gal4 ase-gal80; UAS-mCD8:GFP/UAS-ns3-RNAi*. (C) Quantifications. n = 5 brain lobes. Error bars indicate s.d. *: p < 0.05. Scale bars: 10 μm.

To test whether *ns3* RNAi induces NB proliferation arrest via nuclear import of Pros, we examined type II larval brain NBs. Type II NBs have undetectable levels of Pros, which is thought to allow newborn INPs to remain proliferative (Bayraktar et al., 2010). If nuclear Pros is a required step in *ns3* RNAi induced NB arrest, the type II NBs should show no *ns3* RNAi phenotype. We used a type II NB-specific driver (wor-gal4, ase-gal80) to express *ns3* RNAi and drive expression of GFP in each type II NB lineage. In wild type, the type II NBs were Dpn^+^ Pros-negative and could be labeled by a pulse of EdU, showing that they are proliferative (Fig. 5A, quantified in C). In contrast, *ns3* RNAi type II NBs were Dpn^+^ nuclear Pros-negative, but failed to efficiently incorporate EdU (Fig. 5B, quantified in C). We conclude NS3 knockdown can produce NB proliferation arrest using a Pros-independent pathway. This does not rule out a role for nuclear Pros in type I NB proliferation arrest, but it does show that at least one additional mechanism must be used to block proliferation in *ns3* RNAi type II neuroblasts.

**Fig. 6.**
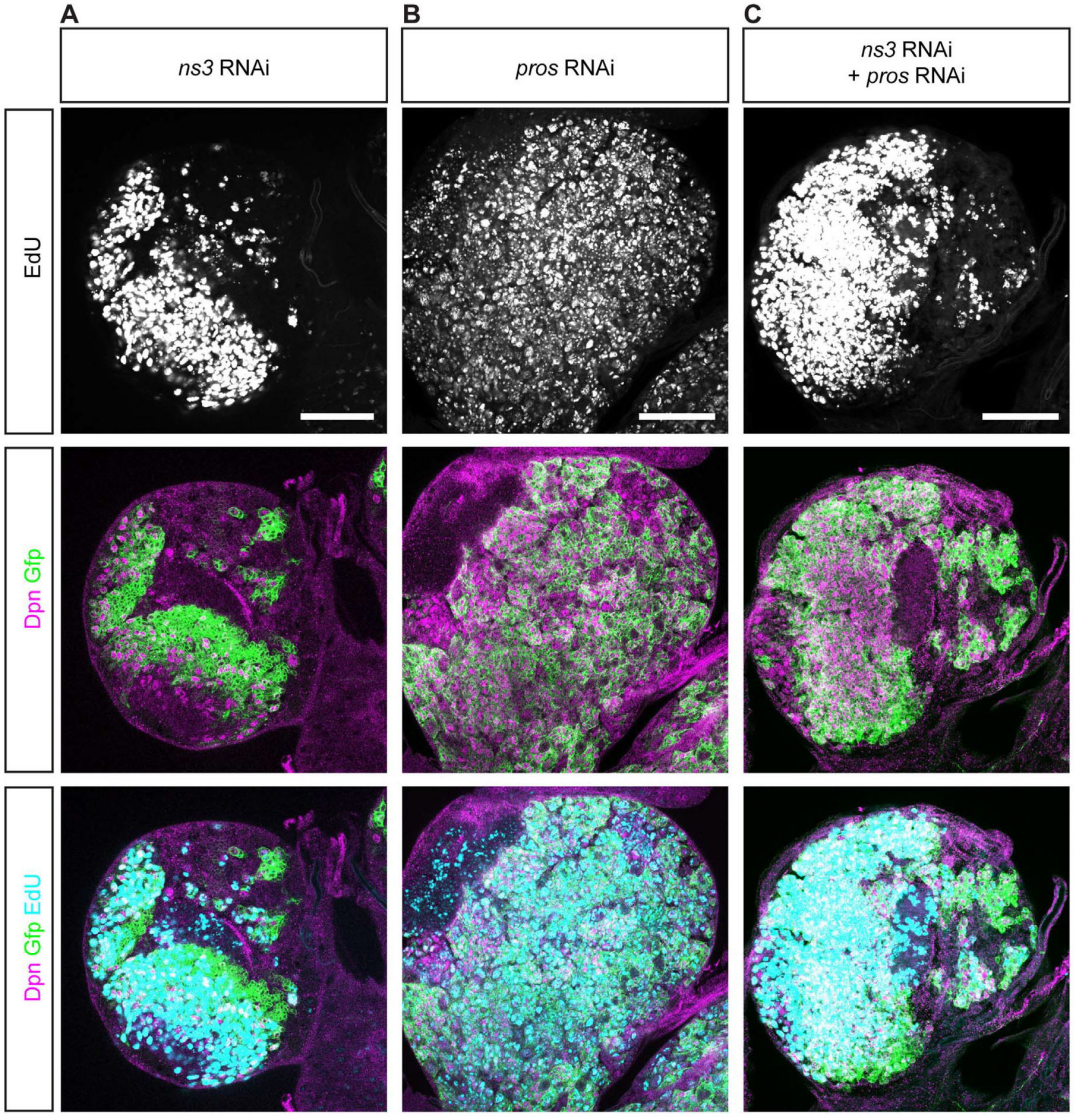
*ns3* pros double knockdown shows intermediate phenotype between *ns3* or pros single knockdown. Single slice confocal images of single or double knockout L3 brains; NBs are identified by Dpn and NBs and their lineages are marked with GFP (*wor-gal4, UAS-mCD8:GFP*). Representative brains are shown (n = 5 brains per genotype). **(A)** *ns3* RNAi brain has fewer EdU+ cells. Genotype: *UAS-Dcr-2; wor-gal4; UAS-mCD8:GFP/UAS-ns3-RNAi*. **(B)** *pros* RNAi brain has many more EdU+ cells. Genotype: *UAS-Dcr-2; wor-gal4/UAS-pros-RNAi; UAS-mCD8:GFP*. **(C)** *ns3* pros double RNAi brain has intermediate number of EdU+ cells. Genotype: *UAS-Dcr-2; wor-gal4/UAS-pros-RNAi; UAS-mCD8:GFP/UAS-ns3-RNAi*. Scale bar: 50 μm.

To determine if *ns3* RNAi also acts via a Pros-independent mechanism in type I NBs, we generated *ns3* pros double RNAi knockdown larvae. If NS3 acts via a Pros-independent mechanism, we would expect that *ns3* pros double RNAi knockdown would match the *ns3* single RNAi phenotype. We found that the *ns3* pros double RNAi knockdown phenotype was intermediate: few proliferating NBs in *ns3* RNAi brains (Fig. 6A), many proliferating NBs in pros RNAi brains (Fig. 6B), and an intermediate level of proliferating NBs in the *ns3* pros double RNAi brains (Fig. 6C). The intermediate phenotype could be due to (a) partial knockdown of both NS3 and Pros proteins, resulting in expansion of the small number of “escaper” NBs that maintain proliferation in *ns3* RNAi brains, or (b) less effective *ns3* RNAi knockdown due to an additional UAS-transgene in the double versus the single knockdown experiment (the extra UAS-transgene may titrate Gal4 levels, thereby reducing expression of all UAS-transgenes). Thus, this experiment did not allow us to determine whether proliferation arrest of *ns3* RNAi type I NBs is Pros-dependent or Pros-independent; both may contribute to the phenotype (see Discussion). Nevertheless, based on our findings that *ns3* RNAi can arrest proliferation of type II NBs in the absence of detectable Pros, we favor the model that NS3 acts via a Pros-independent pathway to maintain NB proliferation in all larval NBs.

### 3.5. NS3 is required to establish NB cortical asymmetry

**Fig. 7.**
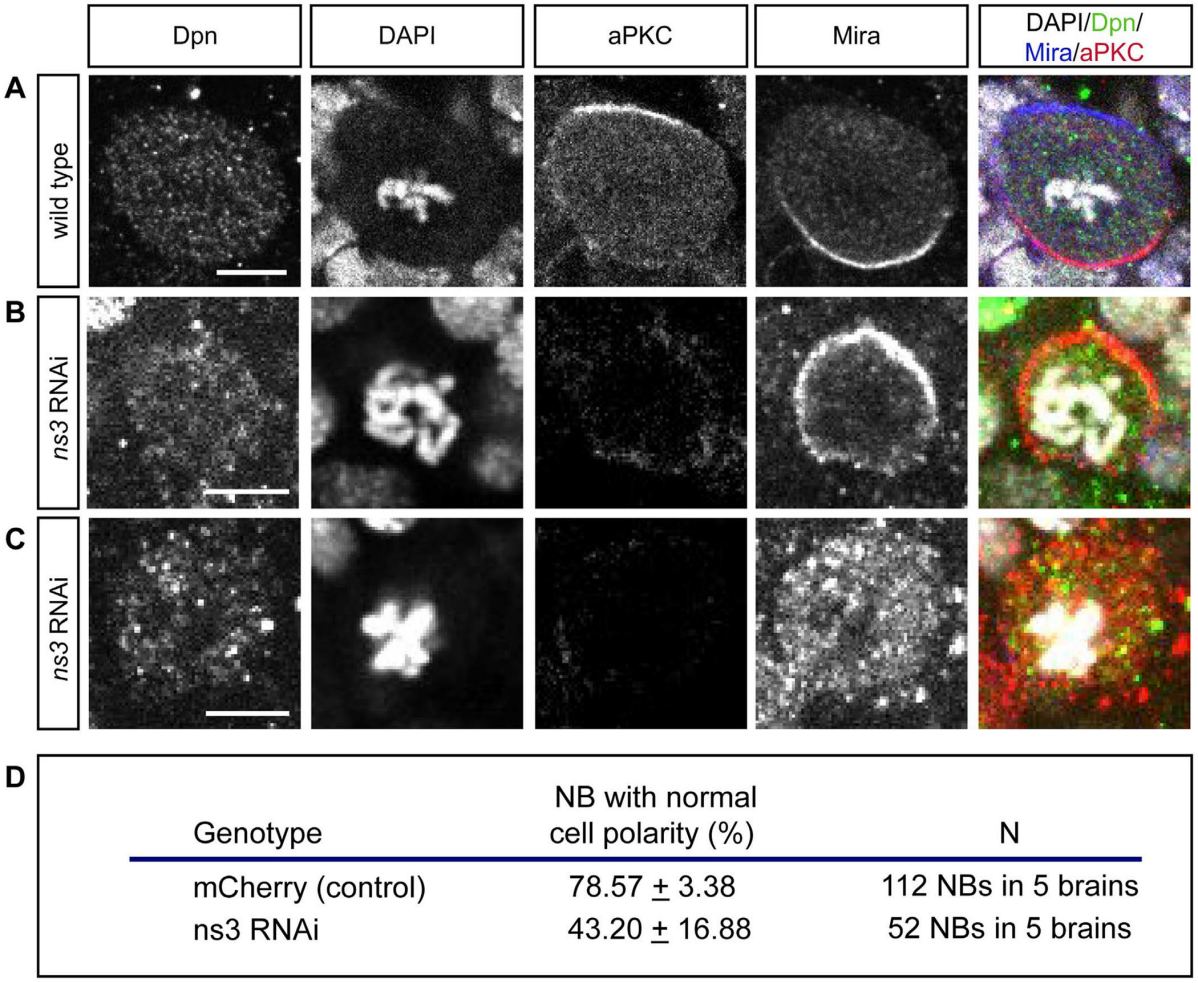
*ns3* RNAi NBs have abnormal cortical polarity. (A) Wild type pro-metaphase NB in the L3 brain, identified by Dpn and condensed chromatin (DAPI). aPKC and Mira asymmetrically segregate to the apical and basal cortex, respectively. Genotype: *wor-gal4; UAS-mCherry-RNAi*. (B,C) *ns3* RNAi pro-metaphase NBs in L3 brain lack detectable aPKC and show either uniform cortical Miranda (Mira; B) or cytoplasmic Mira (C). Genotype: *wor-gal4; UAS-ns3-RNAi*. Scale bars: 5 μm. (D) Quantification of the percent of mitotic NBs that display proper asymmetric segregation of aPKC and Mira.

If NS3 acts via a Pros-independent mechanism, regulating NB cortical polarity is a good possibility, as loss of cortical polarity has been shown to lead to both NB proliferation arrest (Rolls et al., 2003) and nuclear Pros localization (Ikeshima-Kataoka et al., 1997; Shen et al., 1997). Wild type larval NBs segregate Miranda and its cargo protein Pros to the basal cortex during mitosis, and aPKC to the apical cortex, where it excludes the Miranda:Pros complex via phosphorylation of Miranda (Atwood and Prehoda, 2009). Loss of functional aPKC results in delocalization of Miranda, NB cell cycle arrest (Rolls et al., 2003) and premature Pros nuclear localization (unpublished results). We confirm that wild type mitotic NBs have strictly non-overlapping cortical crescents of aPKC and Miranda (Fig. 7A, quantified in D). In contrast, *ns3* RNAi mitotic NBs show a significant loss of cortical polarity, with aPKC mostly undetectable and Miranda uniform cortical or cytoplasmic (Fig. 7B,C, quantified in D). These phenotypes show that NS3 is required for normal NB asymmetric cell division, and the Miranda delocalization phenotype is consistent with failure to segregate Pros out of the NB during asymmetric division, which may lead to abnormal nuclear Pros in the NB following cell division (see Discussion). Our results support a model in which NS3 regulates NB cortical polarity to maintain NB proliferation; note that this does not exclude a role for NS3-regulated Pros nuclear export in maintaining type I NB proliferation.

### 3.6. Defining the NS3 domains required for promoting NB proliferation

**Fig. 8.**
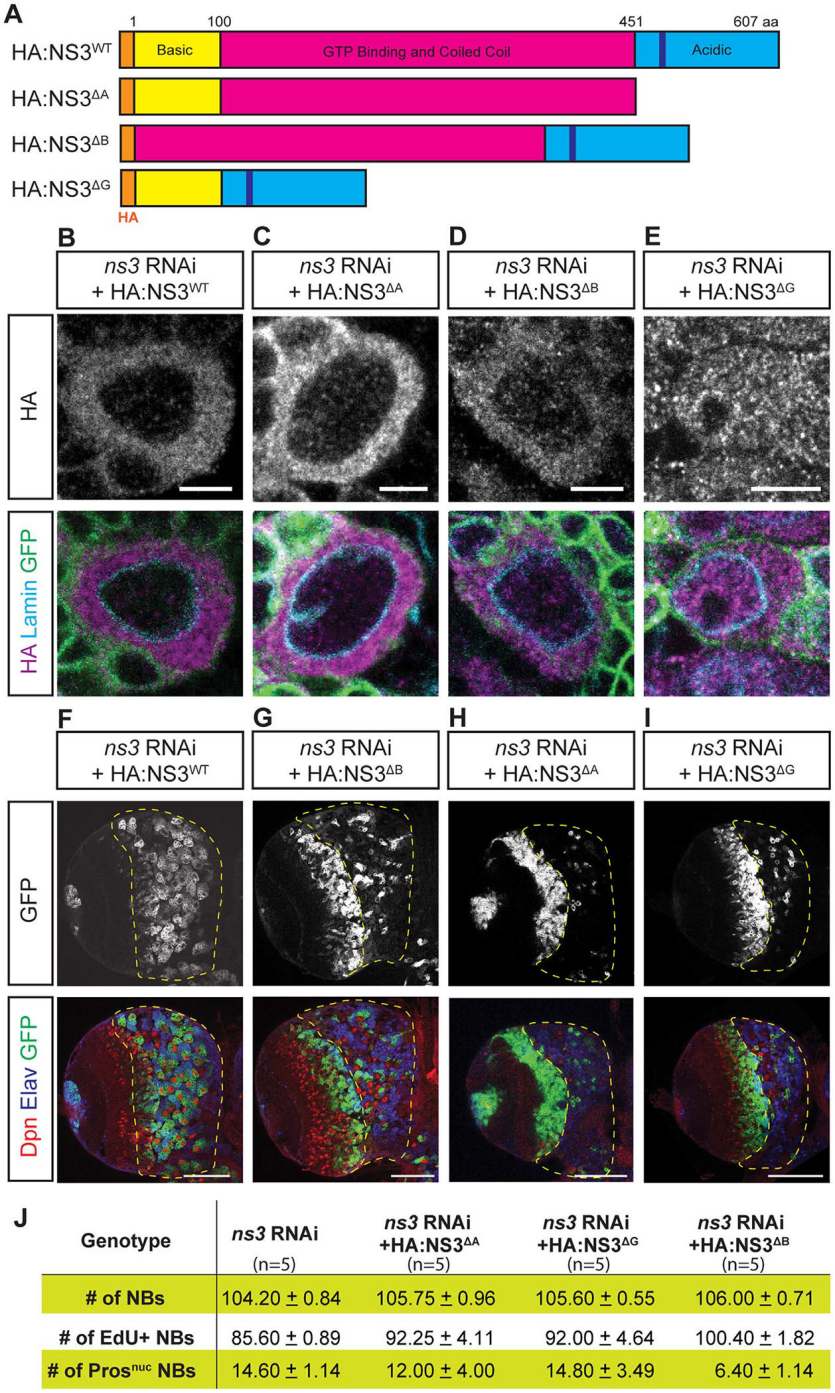
The GTP-binding domain and acidic domain together is essential to promote NB proliferation. (A) Schematics of NS3 and the rescue constructs. The DNA sequence in the acidic domain is mutated to avoid RNAi knocked-down (see Methods). (B-E) L3 brain NBs. The NB-specific driver *wor-gal4* was used to drive *UAS-ns3-RNAi*, the indicated *UAS-HA:NS3* rescue constructs, and *UAS-mCD8:GFP*. HA staining shows NS3 localization, GFP marks the NB membrane, and Lamin marks the nuclear envelope. Genotype: *UAS-Dcr-2; wor-gal4/UAS-NS3 rescue constructs; UAS-mCD8:GFP/UAS-ns3-RNAi*. Scale bars: 5 μm. (F-J) A single focal plane of L3 brain. *ns3*-RNAi and the rescue constructs (F-I) were concurrently expressed by *wor-gal4*, and the cells were marked by *UAS-mCD8:Gfp*. The central brain region was outlined with yellow dash lines. Quantification results (J) from 5 brain lobes. Genotype: *UAS-Dcr-2; wor-gal4/UAS-NS3 rescue constructs; UAS-mCD8:GFP/UAS-ns3-RNAi*. Scale bars: 50 μm.

To understand how NS3 promotes NB proliferation, we performed a structure-function analysis to identify the NS3 protein domains required for this function. NS3 consists of three major domains, the N-terminal basic domain, the GTP binding and coiled-coil domain, and the C-terminal acidic domain (Fig. 8A). The GTP binding domain functions as GTPase and is essential for global body growth (Hartl et al., 2013), yet less is understood about the functional significance of both the basic and acidic domains. To determine the function of each domain, we depleted endogenous NS3 using RNAi and rescued with one of the following constructs: acidic domain-deleted NS3 (NS3^ΔA^); the GTP-binding domain-deleted NS3 (NS3^ΔG^); or the basic domain deleted NS3 (NS3^ΔB^) (Fig. 8A). We also mutated the DNA sequence in the acidic domain to prevent RNAi from targeting the rescue constructs (see Methods).

We co-expressed *ns3*-*RNAi* and each of the four rescue constructs in larval NBs and determined the subcellular localization of the rescue constructs. We found that NS3^ΔA^, and NS3^ΔB^ were located in cytoplasm similar to wild type NS3 localization (Fig. 8B–D); in contrast, the NS3^ΔG^ protein lacking the GTP-binding domain was evenly distributed in both the cytoplasm and the nucleus (Fig. 8E). Expression of the rescue constructs in wild type (without *ns3* RNAi) did not generate any phenotypes (data not shown). We then assayed the ability of each rescue construct to restore proliferation to *ns3* RNAi larval NBs (Fig. 8F–J, quantified in J). Partial rescue was obtained by the NS3^ΔB^ construct (Fig. 8G). In contrast, the NS3^ΔA^ and NS3^ΔG^ constructs failed to rescue the *ns3* RNAi phenotype (Fig. 8H,I). We conclude that the NS3 acidic domain and GTP-binding domain are essential for maintaining larval NB proliferation.

## 4. Discussion

Our genetic screen identified 11 genes, including *ns3*, that are required to prevent premature NB cell cycle exit. These genes include RNA or ribonucleoprotein binding proteins (*CG10418, ns1, ns3 and CG5033*), nuclear-cytoplasmic transport machinery (*Ran, RanGAP, Trn-SR, and Cse-1*), an ATPase (*CG4908*), a calcium signal transducer (*Calmodulin*), and a nuclear protein (*bys*) (Fig. 1A). Our results indicate that multiple pathways are required to promote cell cycle progression. It is unknown yet how each gene contributes to the prevention of NB cell cycle arrest. For example, Calmodulin is highly enriched in NBs (Kovalick and Beckingham, 1992), and we have shown it is required to maintain NB proliferation. Calmodulin is activated by calcium binding and subsequently activates target kinases or phosphatases, but how these targets maintain NB proliferation remains to be explored.

Here we focused on *ns3*. This gene is not expressed at detectable levels in the embryonic CNS (BDGP in situ homepage: http://insitu.fruitfly.org/cgi-bin/ex/report.pl?ftype=10&ftext=FBgn0266284). Consistent with this observation, *ns3* homozygous mutant embryos showed no detectable NB proliferation defect, nor did overexpression of *ns3* in otherwise wild type embryos produce a NB proliferation phenotype (data not shown). *ns3* homozygous mutants failed to grow after larval hatching, possibly due to non-neuronal phenotypes or background lethal mutations, preventing us from analyzing larval NBs. Mosaic clonal analysis of *ns3* mutants in larval NBs did not reveal a NB proliferation phenotype (data not shown), possibly because the strongest available allele is not a null allele (Hartl et al., 2013).

Lsg1, the ortholog of NS3, is a highly-conserved protein and is found in Plantae, Fungi and Animalia, suggesting widespread importance. Lsg1 was identified as required to promote the release of nuclear export adapter from the large ribosomal unit for ribosome final maturation (Hedges et al., 2005; Kallstrom et al., 2003; Reynaud et al., 2005; Weis et al., 2014). NS3 is also required for ribosome biogenesis in the cytoplasm and regulates the synthesis of ribosomal proteins RpL13 and Rps6 (Hartl et al., 2013). Therefore, we had originally hypothesized that NS3 might inhibit the NB cell cycle by down-regulating NB-specific genes that are required for NB proliferation or self-renewal. However, we observed normal expression of many NB-specific markers (Fig. 3), which suggests a distinct mechanism may operate in place of, or in parallel to, a cell growth pathway.

In *Drosophila*, NS3 functions non-autonomously to regulate insulin signaling in dopaminergic neurons to control whole body growth, and expression of insulin downstream effector Akt1 in *ns3* mutants is sufficient to rescue the developmental defect (Kaplan et al., 2008). We found that the cell cycle defect of *ns3* RNAi NBs can be fully rescued by overexpressing NS3 in NBs, showing that NS3 functions cell-autonomously to promote cell cycle progression. Interestingly, reactivation of quiescent NBs and cell cycle progression of proliferating NBs both require Akt1 (Chell and Brand, 2010; Cheng et al., 2011; Sousa-Nunes et al., 2011), and it would be important to know if Akt1 is also downstream of *ns3* pathway in NBs and if *ns3* RNAi compromises production or activation of Akt1.

NS3 has physical interactions with insulin/mTOR signaling components Gigas, Thor, Rheb (Ras homolog enriched in brain) and Myc (Vinayagam et al., 2016), and yet little is known how NS3 regulates insulin/mTOR signaling pathways via interacting with these proteins. More work will be needed to understand if *ns3* RNAi attenuates insulin/mTOR signaling pathways to induce NB cell cycle arrest. Other genes, including ribosomal proteins, that are affected in *ns3* RNAi mutant NBs can be identified in the future by identifying NB-specific RNAs for global transcriptome analysis (Hida et al., 2017; Liu et al., 2015; Miller et al., 2009; Yang et al., 2016).

At the onset of our experiments, it seemed likely that NS3 would function in Ran-dependent transport, because RNAi depletion of NS3, Ran, RanGAP and RanGEF all induce nuclear Pros localization. Surprisingly, we found that the cellular distribution of NLS:NES:GFP reporter was completely different in *Ran* and *ns3* RNAi mutant NBs (Fig. 4), strongly indicating that NS3 and Ran act in the different pathways. This raises the question: why does loss of both Ran and NS3 lead to elevated nuclear Pros? One explanation could be that the Ran pathway acts as a secondary backup mechanism. For example, CRM1 has a pivotal role in relocating proteins “misplaced” in the nucleus, which can frequently occur due to leaky nuclear pore complexes or damage to the nuclear envelope (Fornerod et al., 1997; Fukuda et al., 1997). The Pros NES shows sensitivity to the drug leptomycin B, which is characteristic of CRM1-dependent NESs (Bi et al., 2003; Demidenko et al., 2001). The yeast and human ortholog of NS3, Lsg1, is required for shuttling the nuclear export adapter between cytoplasm and nucleus, and loss of Lsg1 impairs the recycle of nuclear export adapter into nucleus. As such, it is tempting to hypothesize Pros is primarily shuttled to the cytoplasm via the same NS3-dependent nuclear export adapter, and Ran acts as a corrective mechanism. Similarly, it is possible that Pros is regulated by both a Ran-independent and Ran-dependent mechanism.

It is important to note that the Pros NES is currently considered to have an atypical sequence, but the NLS-NES-GFP we used has a canonical NES (Bi et al., 2003; Demidenko et al., 2001). We are beginning to understand NESs are quite diverse and depend not only on the sequence, but also their tertiary structure and physical properties (Xu et al., 2012). Based on the ability to bind CRM1, multiple new consensus sequences have been identified (Kosugi et al., 2008; Xu et al., 2012). Because Pros appears to be CRM1-dependent, we believe the current understanding of what constitutes a consensus sequence simply remains too restrictive, and the use of a canonical NES was not relevant to the Pros localization mechanism.

It has previously been shown that loss of the aPKC cortical polarity protein can lead to NB cell cycle arrest (Rolls et al., 2003). Thus, failure to express, stabilize, or properly localize aPKC to the apical cortex of the mitotic NB appears to be the primary or earliest defect in *ns3* RNAi NBs. It is also possible that there are multiple defects in *ns3* RNAi NBs that cause cell cycle arrest; in type I NBs one defect is abnormal nuclear localization of Pros, which is known to arrest the NB cell cycle. However, at least one other mechanism is utilized by type II NBs, which do not contain detectable levels of Pros (in wild type or *ns3* RNAi NBs). This second mechanism may be failure to properly localize aPKC, or a completely unknown mechanism. Addressing the role of NS3 in type II NBs is an interesting question for the future.

## Supplementary Material

table
