# Supplementary material for: Drosophila nucleostemin 3 is required to maintain larval neuroblast proliferation
Source: Dev Biol. Author manuscript; Available in PMC 2018 Dec 4. (PMC6278609; doi:10.1016/j.ydbio.2018.04.014)
Supplement: table [file NIHMS996547-supplement-table.docx]

**Table S1**

The list of RNAi transgenic flies used in genetic screen. y^1^

| **Gene** | **Genotype** | **Stock Number** |
| --- | --- | --- |
| *Atet* | y^1^ v^1^; P{TRiP.GLC01683}attP2 | 50563 |
| *Br140* | y^1^ v^1^; P{TRiP.HMJ02067}attP40 | 42502 |
| *bys* | y^1^ sc^*^ v^1^; P{TRiP.HMS00196}attP2 | 34876 |
| *cam* | y^1^ sc^*^ v^1^; P{TRiP.HMS01318}attP2 | 34609 |
| *Cse1* | y^1^ v^1^; P{TRiP.JF02972}attP2 | 28337 |
| *CG10418* | y^1^ sc^*^ v^1^; P{TRiP.HMC03933}attP40 | 55746 |
| *CG2875* | y^1^ v^1^; P{TRiP.JF02495}attP2 | 29333 |
| *CG4908* | y^1^ v^1^; P{TRiP.HMC03437}attP40 | 51863 |
| *CG5033* | y^1^ sc^*^ v^1^; P{TRiP.HMS02840}attP40 | 44118 |
| *CG5033* | y^1^ sc^*^ v^1^; P{TRiP.HMS02623}attP40 | 42930 |
| *CG5589* | y^1^ sc^*^ v^1^; P{TRiP.HMS00325}attP2 | 32334 |
| *CG6512* | y^1^ sc^*^ v^1^; P{TRiP.HMS01331}attP2 | 34343 |
| *CG7637* | y^1^ sc^*^ v^1^; P{TRiP.HMC03890}attP40 | 55194 |
| *CG7933* | y^1^ sc^*^ v^1^; P{TRiP.HMS02628}attP40 | 42935 |
| *cycE* | y^1^ sc^*^ v^1^; P{TRiP.HMS00060}attP2 | 33654 |
| *Dl* | y y^1^ sc^*^ v^1^; P{TRiP.HMS01309}attP2 | 34322 |
| *Imp* | y^1^ sc^*^ v^1^; P{TRiP.HMS01168}attP2 | 34977 |
| *Kap-α3* | y^1^ v^1^; P{TRiP.JF02686}attP2 | 27535 |
| *l(2) 37Cc* | y^1^ sc^*^ v^1^; P{TRiP.HMS00702}attP2 | 32912 |
| *Letm 1* | y^1^ sc^*^ v^1^; P{TRiP.HMS01644}attP40 | 37502 |
| *mbf1* | y^1^ v^1^; P{TRiP.HM05036}attP2 | 28550 |
| *mCherry* | y^1^ sc^*^ v^1^; P{VALIUM20-mCherry}attP2 | 35785 |
| *msk* | y^1^ v^1^; P{TRiP.HMS00020}attP2 | 33626 |
| *ns1* | y^1^ v^1^; P{TRiP.JF03301}attP2 | 29622 |
| *ns3* | y^1^ sc^*^ v^1^; P{TRiP.GL00586}attP2 | 36626 |
| *Ran* | y^1^ sc^*^ v^1^; P{TRiP.HMS02885}attP2 | 44587 |
| *RanGAP* | y^1^ v^1^; P{TRiP.JF03244}attP2/TM3, Sb[1] | 29565 |
| *Roe1* | y^1^ sc^*^ v^1^; P{TRiP.HMS02777}attP40 | 44060 |
| *Trn-SR* | y^1^ v^1^; P{TRiP.JF02010}attP2 | 25988 |
| *Unr* | y^1^ sc^*^ v^1^; P{TRiP.HMS00428}attP2 | 32432 |

**Table S2**

The list of primers used to generate NS3 truncation constructs

| Targeted region | Primer sequence | | Fragment size (bp) |
| --- | --- | --- | --- |
| (1) HA-basic-GTP binding-acidic | F | AACTCTGAATAGGGAATTGGATGTACCCCTACGATGTCCCCGATTACGCCATGGGCAAAAAGAACAAGGGCG | 1519 |
|  | R | TAATCCTTTAATACGTATCGTGCGGAACGCGCCTGAT |  |
| (2) Acidic-stop | F | ACGATACGTATTAAAGGATTACGTCAATGGCAGACTGCTCTACG | 409 |
|  | R | CTTCACAAAGATCCTCTAGATCAGTGCTCGTCCAGGTGC |  |
| (3) HA-basic-GTP binding domain-stop | F | AACTCTGAATAGGGAATTGGATGTACCCCTACGATGTCCCCGATTACGCCATGGGCAAAAAGAACAAGGGCG | 1413 |
|  | R | CTTCACAAAGATCCTCTAGATTAGTCCTCGCCTTCCAGCG |  |
| (4) HA-GTP binding-acidic | F | AACTCTGAATAGGGAATTGGATGTACCCCTACGATGTCCCCGATTACGCCCGCATGCACCAGAAGCAC | 1222 |
|  | R | TAATCCTTTAATACGTATCGTGCGGAACGCGCCTGAT |  |
| (5) HA-basic | F | AACTCTGAATAGGGAATTGGATGTACCCCTACGATGTCCCCGATTACGCCATGGGCAAAAAGAACAAGGGCG | 364 |
|  | R | GAATGCGGTGGCCTTTCCATCTGCTCCTGCGTCTTGCTCA |  |
| (6) Acidic | F | TGAGCAAGACGCAGGAGCAGATGGAAAGGCCACCGCATTC | 134 |
|  | R | TAATCCTTTAATACGTATCGTGCGGAACGCGCCTGAT |  |

HA:NS3^WT^ = (1) + (2)

HA:NS3^ΔA^ = (3)

HA:NS3^ΔB^ = (4) + (2)

HA:NS3^ΔG^ = (5) + (6) + (2)
